# Impaired autophagy activity is linked to elevated ER-stress and inflammation in aging adipose tissue

**DOI:** 10.18632/aging.101083

**Published:** 2016-10-24

**Authors:** Amiya Kumar Ghosh, Theresa Mau, Martin O'Brien, Sanjay Garg, Raymond Yung

**Affiliations:** ^1^ Division of Geriatric- and Palliative Medicine, Department of Internal Medicine, University of Michigan, Ann Arbor, MI 48109, USA; ^2^ Geriatric Research, Education and Clinical Care Center (GRECC), VA Ann Arbor Health System, University of Michigan, Ann Arbor, MI 48109, USA

**Keywords:** aging, adipose tissue, inflammation, ER-Stress, autophagy

## Abstract

Adipose tissue dysfunction in aging is associated with inflammation, metabolic syndrome and other diseases. We propose that impaired protein homeostasis due to compromised lysosomal degradation (micro-autophagy) might promote aberrant ER stress response and inflammation in aging adipose tissue. Using C57BL/6 mouse model, we demonstrate that adipose tissue-derived stromal vascular fraction (SVF) cells from old (18-20 months) mice have reduced expression of autophagy markers as compared to the younger (4-6 months) cohort. Elevated expressions of ER-stress marker CHOP and autophagy substrate SQSTM1/p62 are observed in old SVFs compared to young, when treated with either vehicle or with thapsigargin (Tg), an ER stress inducer. Treatment with bafilomycin A1 (Baf), a vacuolar-type H (+)-ATPase, or Tg elevated expressions of CHOP, and SQSTM1/p62 and LC-3-II, in 3T3-L1-preadipocytes. We also demonstrate impaired autophagy activity in old SVFs by analyzing increased accumulation of autophagy substrates LC3-II and p62. Compromised autophagy activity in old SVFs is correlated with enhanced release of pro-inflammatory cytokines IL-6 and MCP-1. Finally, SVFs from calorie restricted old mice (CR-O) have shown enhanced autophagy activity compared to *ad libitum* fed old mice (AL-O). Our results support the notion that diminished autophagy activity with aging contributes to increased adipose tissue ER stress and inflammation.

## INTRODUCTION

Chronic low-grade inflammation is a hallmark of aging that plays a crucial role in many age associated diseases [1, 2]. White adipose tissue (WAT) is recognized as a major source of chronic pro-inflammatory cytokines in aging [3, 4]. These cytokines promote insulin resistance and contribute to the development of type-2 diabetes (T2D) and other age-related diseases [5, 6]. However, the detailed molecular and cellular mechanisms that promote aging adipose tissue inflammation are poorly understood.

Adipose tissue can be broadly separated into adipocytes and the adipose tissue stromal vascular fraction (SVF). The main cellular constituents of SVF include pre-adipocytes, endothelial cells, immune cells and other cells. Adipose tissue SVFs has also been demonstrated to have an inflammatory profile associated with aging and obesity [3, 4, 7]. Although a higher number of inflammatory M1 macrophages has been correlated with aging adipose tissue inflammation [3, 4, 8], characterization of adipose tissue cellular fractions [9, 10] has shown that preadipocytes of SVFs and are a pre-dominant source of adipose tissue pro-inflammatory cytokines. Preadipocytes are one of the largest progenitor pools (≈15-50% in fat depots) in the body and harbor following characteristics [9]. a) Preadipo-cytes replicate in response to mitogens, including IGF-1; b) Metabolic and secretory profiles of preadipocytes are distinct from differentiated fat cells, and vary among fat depot; c) Preadipocytes express Toll-like receptors (TLRs) and have full innate immune response capabilities; d) The gene expression profile of pre-adipocytes is closer to macrophages than fat cells; e) Activated preadipocytes can also acquire macrophage-like morphology phenotypes.

Extensive studies from James Kirkland's group and others suggested that changes in preadipocyte function during aging lead to dysfunctional adipose tissue, eventually progressing to chronic inflammation [9]. These changes include the decline preadipocyte replication, decreased adipogenesis, increased susceptibility to lipotoxicity and increased pro-inflammatory cytokines, chemokines, extracellular matrix (ECM)-modifying protease and stress response element expression. We have recently demonstrated that elevated endoplasmic reticulum (ER) stress response contributes to greater inflammatory response [11] in aging adipose tissue SVFs. However, the molecular events that lead to its increased susceptibility to ER stress response in aging adipose tissue are poorly defined.

We speculated that elevated ER stress in aging adipose tissue may be due to defective protein turnover by proteasomes and/or lysosomes. Recent reports suggested that the macro-autophagy (hereafter autophagy) pathway, an evolutionarily conserved cellular protein degradation mechanism, contributes to ER stress [12, 13]. Autophagy is a catabolic process for autophagosome-lysosomal degradation of bulk cytoplasmic content of proteins. There are three major steps that regulate autophagy [14] a) *induction*: in response to ‘nutrient deprivation’, phosphatidylinositol 3-kinase (PI3K) activates the mammalian target of rapamycin (mTOR), which in turn, blocks autophagy through inhibition of AuTophaGy related (Atg) 1 from recruiting its partners Atg13 and Atg17, b) *Vesicle nucleation*: is mediated by Atg6/Beclin-1, which forms a complex with Vps34, the class III PI3K. Beclin-1 is an important interface between autophagy and cell death pathways, by virtue of its binding capacity with anti-apoptotic proteins Bcl2 and Bcl-x_L_, c) *Vesicle elongation*: this process involves Atg3-and Atg7-mediated conjugation of microtubule-associated protein1 light chain 3 (MAP LC3-I) to the membrane lipid phosphatidylethanolamine to form LC3-II. This serves as a recognition site for LC3-binding chaperones such as SQSTM1/p62 that deliver their cargo to auto-phagosomes. Once formed, autophagosomes traffic along microtubules to reach lysosomes where they fuse to form autophagolysosomes, allowing the degradation of their contents by lysosomal acid hydrolases. The implication of autophagy has been demonstrated in many inflammatory diseases that involve ER-stress response, including obesity [15-17], non-alcoholic fatty liver diseases (NAFLD) [18], myopathies [19], and neurodegeneration [20]. The nutrient sensing TOR pathway is well known to affect several crucial cellular functions including protein synthesis and autophagy that modulate aging processes [21]. More importantly, pharmacological agents that inhibit mTOR, and thereby promote autophagy has also been demonstrated to extend health span and lifespan of the organisms. For example, spermidine and resveratrol promote autophagy [22], rapamycin reprogram energy metabolism in old hearts by transiently mitochondrial remodeling in mice [23].

This study was undertaken to test our hypothesis that impaired autophagy in aging adipose tissue SVFs contributes to elevated ER stress and inflammation. We tested the relative expression profile of autophagy-associated genes in adipose tissue-derived SVFs from young and old mice. The relative accumulation of autophagy substrate p62 in the SVFs derived from young and old mice were analyzed following treatment with either vehicle control or with Tg. We also recapitulated the association of autophagy activity and ER-stress using 3T3-preadipocyte cell line treated with either an ER stress inducer (Tg) or the autophagy blocker bafilomycin A1 (Baf). Autophagy activity was measured by determining the levels of p62 and microtubule associated light chain II (MAP LC3-II) in the SVFs lysates from young and old mice treated with Baf *ex vivo*. Productions of inflammatory cytokine were also determined under aforementioned treatment conditions to establish the link between autophagy activity and inflammation in aging SVFs. Finally, we compared autophagy activity in the SVF lysates of old mice (AL-O) compared to calorie restricted old (CR-O) mice, a well-characterized model of slow aging with reduced inflammation.

## RESULTS

### Diminished expression of autophagy-associated genes in aging adipose tissue SVFs

**Figure 1:**
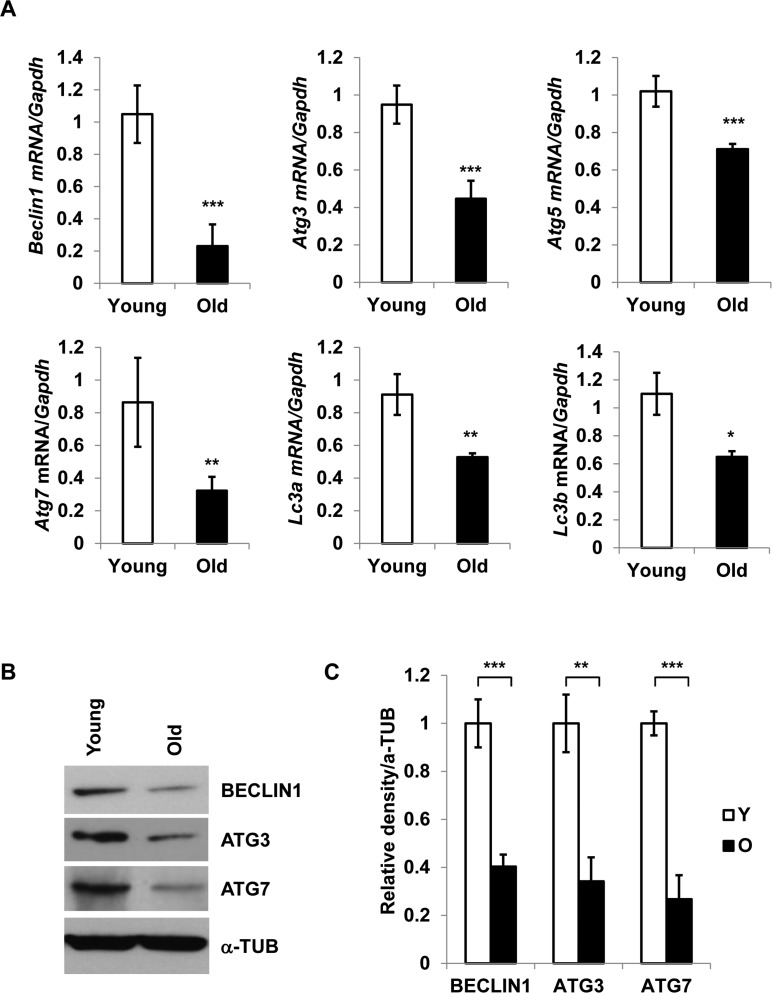
Diminished expression of autophagy gene products in aged SVFs (**A**) Relative mRNA expression of autophagy genes *Beclin1*, *Atg3*, *Atg5*, *Atg7*, *Lc3a* and *Lc3b* in adipose tissue SVF of young (4 m) and old mice (20 m) analyzed by real-time PCR using Ct values. Data represented in bar diagrams are Mean + SD value of relative mRNA expression from three independent experiments where total RNA was extracted from SVFs of young (n=5) and old (n=3) mice and used as a template for one-step RT-qPCR reaction. The significance levels *p<0.05, **p<0.001 or ***p<0.0001 were analyzed by unpaired student's t-test using means and SDs. (**B**) Protein expressions of BECLIN1, ATG3 and ATG7 were analyzed by Western blotting of SVF lysates from young (n=5) and old (n=3) mice. Data presented here are representative image of three independent experiments. The relative density of protein bands were plotted in (**C**). Values were expressed as Mean + SD. of three independent experiments and the significance levels * p<0.05 or **p<0.001 were analyzed by Student's t-test.

To investigate the role of autophagy in adipose tissue inflammation, we focused on the adipose tissue-derived SVFs, as this is the predominant source of pro-inflammatory cytokines [4, 11] in fat. We analyzed mRNA expression of key autophagy-associated genes *Beclin1*, *Atg3, Atg5, Atg7, Lc3a* and *Lc3b* in SVFs derived from epididymal fat pads of young (4-6 months) and old (20 months) mice by RT-qPCR analysis. Significant reduction in mRNA expression of autophagy-associated genes was observed in old SVFs compared to young (Fig. 1A). With the exception of *Beclin1*, we found no difference in the expression of *Atg7*, *Atg3* or *Lc3b* mRNA between young and middle-aged groups (Fig.S1). These data indicated that diminished expression of autophagy genes primarily occurs from middle age onward.

To validate the RT-PCR results, we performed Western blot analysis of Beclin1, Atg3, and Atg7 on SVFs derived from young and old mice (Fig. 1B). The results confirmed diminished protein expression of autophagy-associated proteins in old SVFs (Fig. 1B and 1C). These data suggested that age-associated reduction of autophagy components may compromise the autophagy process, which may in turn result in increased ER stress and elevated pro-inflammatory cytokine production in old adipose tissue.

### Elevated ER-stress induces P62 accumulation in old SVFs

**Figure 2:**
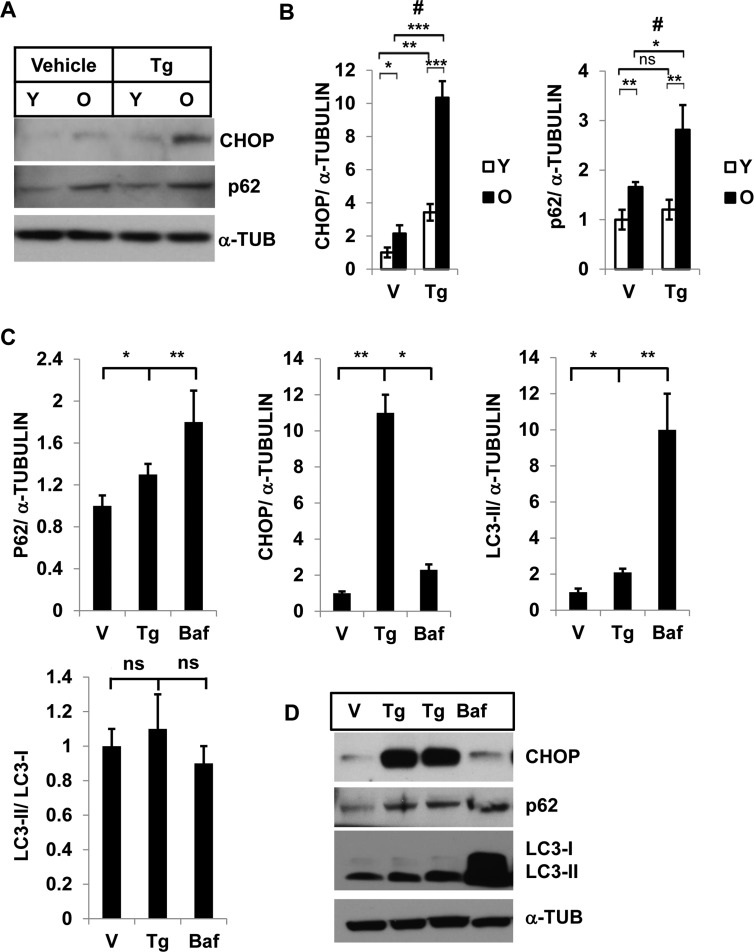
Interaction of autophagy and ER stress response in SVFs from adipose tissue and in 3T3-preadipocytes Different levels of CHOP and SQSTM1/p62 in SVF lysates from young and old mice with vehicle or Tg treatments. **(A**) Western blot analysis of ER-stress response protein CHOP and autophagy associated protein SQSTM1/p62 in the SVF lysates from young (n=5) and old mice (n=3) treated with either vehicle (DMSO) or thapsigargin (30 nM) for 18h. The density of protein bands from three independent experiments were plotted in (**B**). Student's t-test was performed using means and SD where *p<0.05 or **p< 0.01 and ***p<0.001 were designated significance levels. Symbol # indicated significance level (p<0.05) of two-way ANOVA analysis for the interaction between treatments (vehicle or Tg) and age factor (Y vs. O). (**C**, **D**) 3T3-preadipocytes were treated with either vehicle or Tg (30nM) or Baf (10nM) for 18 hrs and the protein levels of p62, CHOP and LC3-II were analyzed by western blots (**D**). The relative density of protein bands for P62, CHOP, LC3-II were plotted (**C**) after normalization with ɑ-Tubulin. Student's t-test was performed and *p<0.05 or **p <0.01 considered significant.

To determine ER-stress and autophagy activity, we treated SVFs from young and old mice with either vehicle or the ER stress inducer Tg, and evaluated the expression levels of p62 and CHOP by Western blotting (Fig. 2A). We found significantly higher levels of both p62 and CHOP in old, compared to young, SVFs treated with either vehicle or Tg. (Fig. 2A, 2B). The accumulation of p62 also paralleled the CHOP expression in old SVFs, suggesting that compromised autophagy correlates with elevated ER stress response in old SVFs.

### ER stress induces STSQM1/p62 accumulation and autophagy block results in elevated ER stress in 3T3-preadipocytes

Since SVFs from adipose tissue consists of mixed population of cells with significant number of preadipocytes (50% of SVFs) [24] we examined the association between autophagy and ER stress response in a pure preadipocyte population. 3T3-preadipocytes were treated with either Tg or Baf and the levels of autophagy substrates p62 and LC3-II, and ER stress protein CHOP expressions were determined by Western blotting analysis (Fig. 2C and 2D). In both treatments, we noticed significant increased accumulation of p62 and LC3-II proteins (Fig.2D). These results confirm that induction of ER stress leads to accumulation of autophagy substrates p62 and LC-3-II. Similarly, significant accumulation of CHOP was observed when 3T3-preadipocytes were treated with the autophagy blocker Baf.

### Autophagy activity is diminished in SVFs from old mice

**Figure 3:**
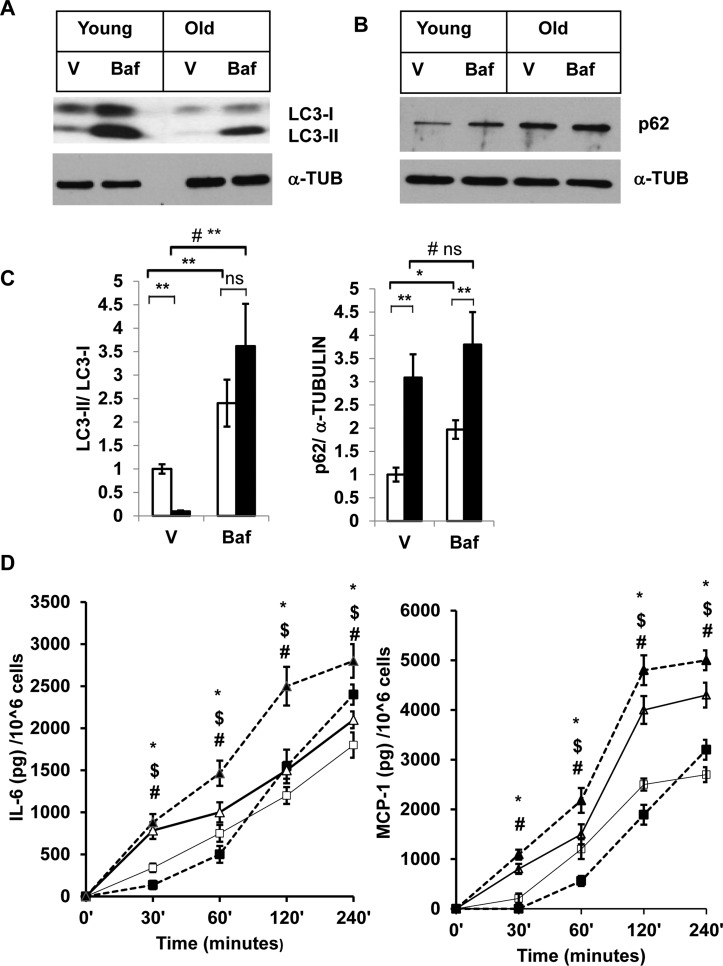
Enhanced accumulation of autophagy substrates in old SVFs and elevated pro-inflammatory cytokines release after autophagy block **(A** and **B**) Western blot analysis of autophagy associated proteins SQSTM1/p62 and LC3-I and LC3-II in SVF lysates from young (n=5) and old mice (n=3) treated with either vehicle (DMSO) or Baf (10 nM) for 18h. The density of protein bands from three independent experiments were normalized with ɑ-tubulin and plotted in (**C**). Student's t-test was performed using means and SD where *p<0.05 or **p<0.01 was considered significant. Symbol # indicated significance level (p<0.05) of two-way ANOVA analysis for the interaction between treatments (vehicle or Tg) and age factor (Y vs. O)**. (D**) Autophagy block results in elevated inflammatory cytokine production in old SVFs. Time dependent production of major pro-inflammatory cytokines (IL-6, MCP-1) by the SVF from young (square box) and old mice (triangle) treated with either vehicle (open) or Baf (filled) were analyzed by ELISA. Values were presented as mean + SD of three independent experiments. Significance of difference between means was determined by Student's t-test and indicated by * p<0.05 for young and ^$^ p<0.05 for old. Symbol ^#^ indicated the significance level (p<0.05) of two-way ANOVA analysis for the interaction between treatment (vehicle and Baf) and age factor (Y vs. O).

We then investigated autophagy activity in SVFs from both young and old mice by treating the cells with either vehicle or Baf, and analyzing the levels of LC3-II, and p62 proteins (Fig. 3A and 3B). We found that young SVFs treated with either vehicle or Baf accumulate higher levels of LC3-II compared to old SVFs when protein bands were normalized to LC3-I (Fig. 3A and 3C). This observation indicates that autophagy activity is compromised in the old SVFs compared to young SVFs. The level of p62 was also higher in aging SVFs treated with either vehicle or Baf compared to young SVFs (Fig. 3B and 3C).

### Blocking autophagy increases SVF inflammatory cytokine production

To corroborate the association between autophagy activities in the SVFs and inflammation, we performed autophagy blocking experiments using SVFs from young and old mice with Baf. We then measured the two major pro-inflammatory cytokines (IL-6 and MCP-1) in the cultured supernatants at different time points after the treatments. We observed significantly higher levels of IL-6 and MCP-1 release by old, compared to young SVFs that are treated with either vehicle or Baf at 30 min, 1h, 2h and 4h (Fig. 3D). Treatment of Baf induced increased cytokine release by old SVFs at all the time points examined. We also noticed an initial decrease in the levels of both IL-6 and MCP-1 from young SVFs upon Baf treatment. However, at 4h, the levels of both cytokines were higher in the Baf-treated young SVFs.

### Autophagy activity is augmented in calorie restricted (CR) mice

**Figure 4:**
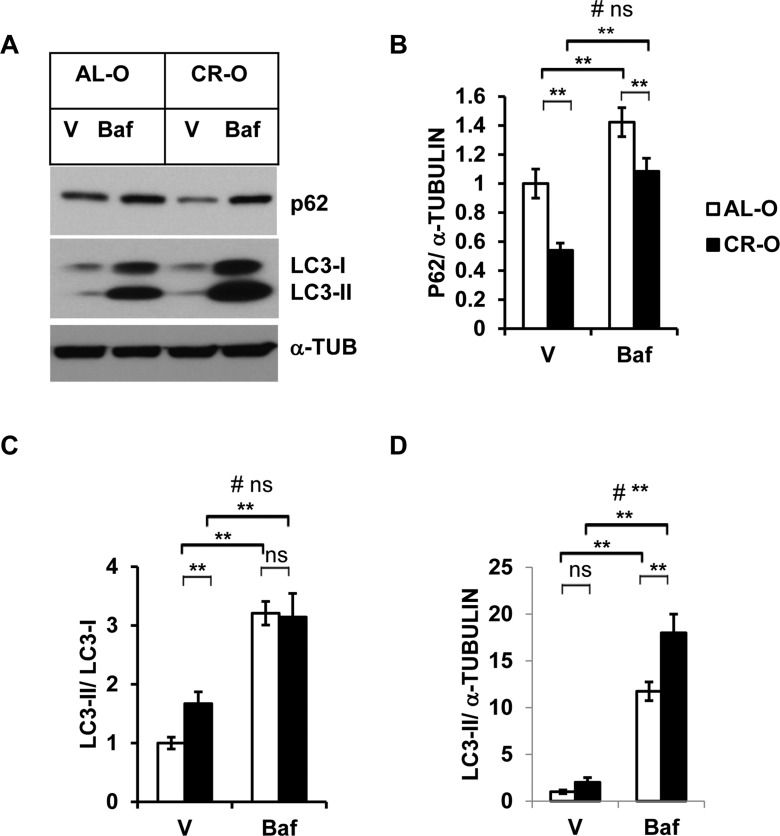
Enhanced autophagy activity in calorie restricted (CR) old mice (**A**) Western blot analysis of autophagy-associated proteins SQSTM1/p62 and LC3-I and LC3-II in the SVF lysates from old (n=3) and old CR mice (n=5) treated with either vehicle or Baf (10 nM) for 18h. The density of protein bands from three independent experiments were normalized with ɑ-Tubulin and plotted in (**B**-**D**). Data presented here are representative of three independent experiments. Values were presented as mean + SD of three independent experiments. ** refers to significance level (p<0.001) of Student's t-test analysis using means and SDs. # indicates the significance level (p<0.05) of two-way ANOVA analysis for the interaction between treatment (Vehicle or Baf.) and aged mice (AL-O vs. CR-O).

Calorie restricted mice are a well-characterized model for extended longevity and are accompanied by delayed onset of multiple age associated diseases [25]. We analyzed autophagy activity in the SVFs from calorie restricted old mice (CR-O) compared to *ad libitum* fed (AL-O) old mice. We found diminished accumulation of autophagy substrate SQSTM/p62 in the SVFs from CR-O mice compared to AL-O mice treated with either vehicle or Baf (Fig. 4A and 4B). The ratio of LC3-II and LC3-I was significantly higher in the SVFs lysates from CR-O mice compared to AL-O mice in the vehicle treated, but not in the Baf-treated samples (Fig. 4C). However, compared to AL-O derived SVFs, there was significantly higher accumulation of LC3-II in the Baf-treated SVFs from CR-O mice, when LC3-II levels were normalized with ɑ-Tubulin. We found significant interaction between these two group of mice (AL-O vs CR-O) and treatments (vehicle vs Baf) when LC3-II levels were normalized with ɑ-Tubulin.

## DISCUSSION

Adipose tissue is at the crossroad of longevity and age-associated diseases involving inflammation and metabolic dysfunction. Excess or dysfunctional fat tissue accelerates the onset of multiple age-related diseases, while interventions that delay or limit fat tissue turn over, redistribution, or dysfunction in experimental animals are associated with enhanced life span. Thus, longevity is extended with interventions that limit visceral fat development, such as: a) caloric restriction [26] b) fat cell insulin receptor knock out (FIRKO), insulin receptor substrate (IRS-1) or S6 kinase 1 knockout mice models [27, 28] c) growth hormone receptor knock out (GHRKO) mice model [29] d) with rapamycin treatment [30, 31] and also by e) surgical removal of visceral fat [32, 33]. The present study was an attempt to investigate the cellular and molecular mechanisms that determine the impact of aging on adipose tissue-derived cells responsible for low-grade systemic inflammation in late-life.

We demonstrated that impaired autophagy activity with aging is linked to elevated ER stress and inflammation in adipose tissue SVFs. The relative expression of autophagy-associated genes (*Atg*) in adipose tissue SVFs from old mice was significantly lower compared to young mice. In addition, autophagy activity in old SVFs was diminished after Tg treatment, as demonstrated by enhanced accumulation of the autophagy substrate SQSTM1/p62. A strong association of autophagy activity and ER-stress was also recapitulated when 3T3-preadipocytes were treated with either ER stress inducer (Tg) or autophagy blocker (Baf). Increased accumulation of SQSTM1/p62 and diminished levels of lipid modified microtubule associated light chain II (MAP LC3-II) was observed in old SVFs treated with vehicle or Baf *ex vivo* (Fig. 3A and 3B), suggested compromised autophagy function in old SVFs compared to their younger counterparts. Elevated production of pro-inflammatory cytokines from the old SVFs under such conditions also established the causal link between autophagy activity, ER stress and inflammation (Fig. 3D).

Caloric restriction (CR) is one of the most robust interventions that delay aging in diverse species including mammals [34, 35]. Emerging evidence suggests that caloric restriction augment autophagy activity in many organs including skeletal muscle [36], liver [37], kidney [38], heart [39], and brain [40] in rodents. In this study, we provide further evidence that caloric restriction promotes autophagy function of adipose tissue SVFs (Fig. 4). Our data strongly suggest that autophagy activity of adipose tissue cells plays a critical role in age-associated adipose tissue ER stress response and inflammation.

**Figure 5:**
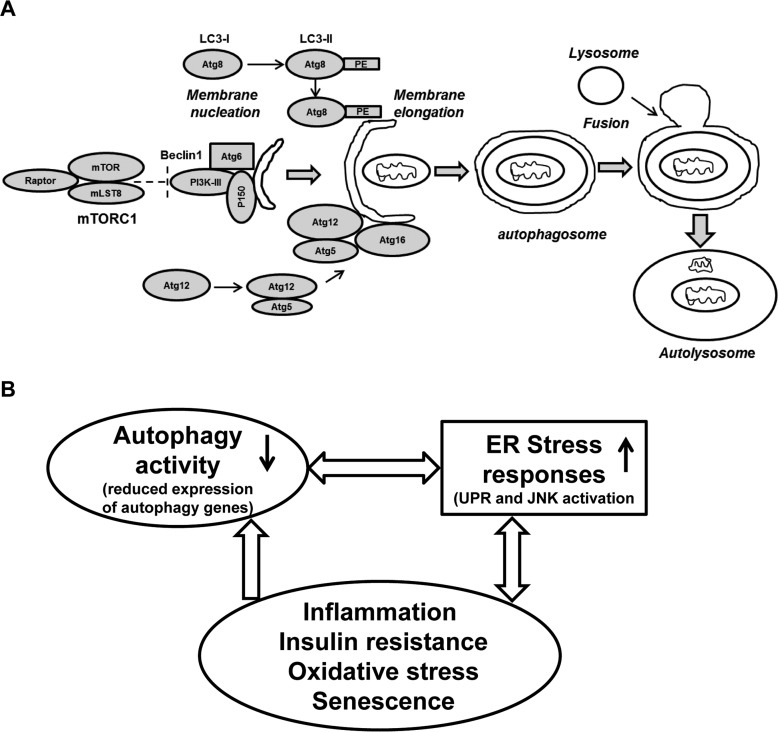
Schematic presentation of the autophagy process and its regulation: Connection with ER stress and oxidative stress and cellular senescence **(A)** The formation of the initial membrane nucleation requires a kinase complex consisting of Beclin 1(Atg6), myristylated kinase (P150) and class III PI3K. The isolation membrane chooses its cargo (in this figure a mitochondria) and elongates until the edges fuse forming a double-membrane structure called an autophagosome. Two ubiquitin-like conjugation systems forming Atg8-PE (LC3-II) and Atg5-Atg12 are necessary for the elongation of the isolation membrane. The autophagosome matures by fusing with lysosomes, finally forming the autolysosomes. Abbreviations: mLST8: mammalian lethal with SEC13 protein 8, PI3KIII: phosphoinositidine 3-kinase class III, PE: phosphatidylethanolamine. **(B)** A simplified model of autophagy activity in aging SVFs: Diminished expression of autophagy machinery results in reduced autophagy activity. Compromised autophagy activity may lead to elevated ER stress, inflammation and oxidative damage resulting senescence. The senescent SVFs in turn further initiates a vicious cycle of compromised autophagy activity, elevated ER stress response and inflammation in the aging adipose tissue.

From our previous study [11] and present study we postulate that compromised chaperoning capacity, along with the diminished expression of autophagy associated components in aging results in accumulation of unfolded or misfolded proteins in the ER. This leads to the Unfolded Protein Response (UPR) or ER stress response. One of the ER stress response pathways involves the activation of c-Jun N-terminal kinase (JNK). Activated JNK (p-JNK) then activates transcription factors NF-κB and AP1. These factors induce the production of pro-inflammatory cytokines and switch on the vicious cycle of inflammation in the aging adipose tissue (Fig. 5A and B).

Our study supports an important role of aberrant autophagy in aging-associated adipose tissue inflammation. This is in agreement with reports on the role of autophagy in other organs in aging and age-associated diseases. For example: expression of the autophagy-related gene Atg7 and LC3-II protein declines, as levels of p62 and polyubiquitin accumulates, concomitant with decreased autophagy in aging rat kidney [41]. Similar phenomena have been documented in liver and thymus of aged mice [42]. Absence of several autophagy related genes resulted in increased aggregates of damaged proteins and organelles in mice [43], abrogated lifespan extension in daf-2 mutants of C. elegans [44], and shortened the lifespan in Drosophila adults of stocks that are hypersensitive to nutrient and oxidative stress [45]. Many of the pathways implicated in the control of lifespan in invertebrates, mice, and humans, including SIRT1, mTOR, Foxo3, NF-κB and P53 pathways, are known to modulate autophagy [46]. Similarly, rapamycin, an activator of autophagy, extends life span from fly to mammals [22, 31, 47], suggesting that manipulation of autophagy may be able to provide insights into the molecular mechanism of aging processes. Fibroblasts from long-lived mutant mice exhibited enhanced autophagy and lower mTOR activity after nutrient deprivation or oxidative stress [48]. Our observation on aging adipose tissue supports an important role of autophagy in the aging process, and links age-related inflammation with an altered ER stress response.

We are beginning to understand the detailed molecular events that led to aging adipose tissue inflammation. Along with our current observations, exciting new data are now linking elevated ER stress response [11] with compromised autophagy and accumulation of senescent cell progenitors [49] as upstream molecular events in adipose tissue inflammation in aging. Our study identifies the interplay of autophagy activity and ER stress response in aging adipose tissue, however the molecular links between two of these pathways requires further investigations.

## MATERIALS AND METHODS

### Mice

C57BL/6 young (4-6 months), middle-aged (10-12 months), old (18–22 months) and calorie restricted (CR) old (18-20 months) male mice were obtained from the National Institute on Aging (NIA) aged rodent colonies. Mice were maintained in a pathogen-free environment at the Unit for Laboratory Animal Medicine (ULAM) facility at the University of Michigan (Ann Arbor, MI) until they were used. All the experimental research in the current study has been approved by the University of Michigan University Committee on Use and Care of Animals (UCUCA).

### Reagents

Thapsigargin (Tg), Bafilomycin A1 (Baf) and Collagenase D were obtained from Sigma-Aldrich (St. Louis, MO). All the chemicals were dissolved in the appropriate media solution or dimethyl sulfoxide (DMSO), as per manufacturer's instructions, and used at the indicated concentrations.

### Isolation of adipose tissue

Careful inspection was done to exclude aged animals with cancer or lymphoma. Gonadal/epididymal fat pads were excised under sterile condition. Fat tissue was fractionated into adipocytes and stromovascular fractions (SVFs), as previously described [11].

### RNA extraction and real-time quantitative PCR (RT-qPCR)

Adipose tissue SVFs were placed directly in RNA lysate buffer and RNA was extracted using the RNeasy kit (Qiagen). RNA was purified using RNeasy Lipid Tissue Midi Kit (Qiagen). Real-time PCR experiments were performed using QuantiTect SYBR green RT-PCR kit (Qiagen) using RNA samples with Corbett Rotor Gene 6000 Series (Qiagen, USA). Data analysis was performed by the comparative 2^**^(−ddCT)^** method using Ct values.

### Cell culture and treatment

Adipose tissue SVFs pellets were re-suspended in DMEM media containing 10% heat-inactivated FBS and plated in 25 cm^2^ flask at the density of 1×10^5^/cm^2^. After 12 hours of incubation in a 37°C/5% CO2 incubator, floating cells were discarded, adherent cells were washed, trypsinized and plated on 12 well plates at a density 5×10^4^cells /cm^2^. Cells were then treated with either vehicle (DMSO), Tg (30nM) or Baf (10nM) and an aliquot of culture supernatant was collected at the indicated time points following the treatments for cytokine assays. For protein analyses, cells were washed with PBS and lysed in RIPA buffer after 18 hrs for protein lysates. Similarly, 3T3-preadipocytes were plated in 6-well plates at density of 1×10^5^/well overnight. The cells were then treated with either DMSO vehicle, Tg (30nM) or Baf (10nM) and harvested in RIPA buffer 18 hrs post-treatment.

### ELISA

IL-6, MCP1 and TNF-ɑ levels in culture supernatants from treated SVFs were measured using Quantikine ELISA kit (R & D Systems, Inc. Minneapolis, USA), according to the manufacturer's protocol.

### Western blotting

Protein quantities were analyzed by standard Western blotting technique. Briefly, 25-50 μg total proteins lysates were separated on Mini PROTEAN Precast Gels with 2 X Laemmli sample buffer (with 2.5% β-mercaptoethanol) to a final 30 μL volume. The proteins were transferred onto a PVDF-membrane and blocked with superblock solution and probed with anti-BECLIN1 (1:1000), anti-ATG3 (1:1000), anti-ATG7 (1:1000) and anti-LC3A/B (1:1000) from Cell Signaling Technology (Danvers, MA); anti-CHOP, anti-p62 (1:500, Santa Cruz Biotechnology), and anti-α Tubulin (1:5000, Abcam) for overnight at 4˚C. Anti-rabbit or anti-mouse horseradish peroxidase (HRP)-conjugated secondary antibodies (Santa Cruz Biotechnology) was used at a dilution of 1:5000 for 1 hr. at room temperature. The binding of specific antibodies was visualized via exposure to a photographic film after treating with enhanced chemiluminescence system reagents (Fisher Scientific, USA). The film was scanned and the band density was quantified by ImageJ (NIH) software. The results were expressed as a relative ratio of the target protein to reference protein.

### Statistical analysis

Results are expressed as Mean + SD in bar diagrams. The significance of difference between means with single variable was analyzed by Student's t-test, and two-way ANOVA analysis was performed with data involving more than two variables to account for the interaction between them. The value of *p* <0.05 was considered to be statistically significant in all statistical analyses. All the statistical tests were performed with Graph Pad Prism 6 software Inc. (La Jolla, CA, USA).

## SUPPLEMENTARY FIGURES


