# Supplementary material for: Impaired autophagy activity is linked to elevated ER-stress and inflammation in aging adipose tissue
Source: Aging (Albany NY). 2016 Oct 24;8(10):2525–36. doi: 10.18632/aging.101083 (PMC5115904; doi:10.18632/aging.101083)
Supplement: Supplementary file 1 [file aging-08-2525-s001.pdf]

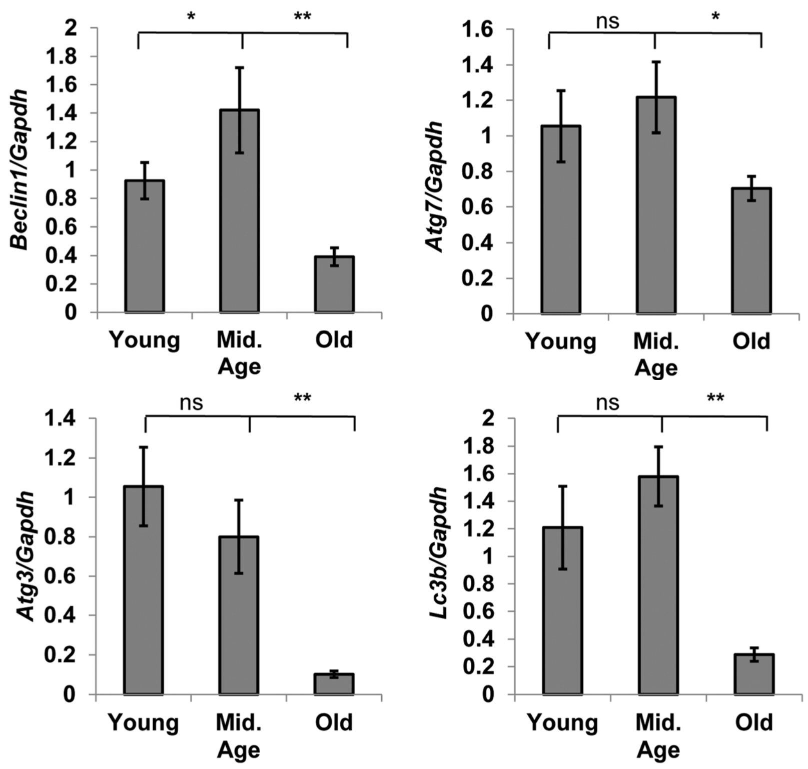

Figure S1. Diminished expression of autophagy associated gene products in SVFs of old mice compared to young and middle aged mice. Relative mRNA expression of autophagy genes *Beclin1*, *Atg7*, Atg3 and *Lc3b* in the SVFs from young (4 m), middle aged (12 m) and old (20 m) mice were analyzed by real-time PCR. Values expressed are Mean± SD of three independent experiments after normalization with respective *Gapdh*. Total RNA extracted from SVFs of young (n=5) and old (n=3) mice were used as template for one step RT-qPCR reaction. The significance of difference between means were indicated as *p<0.05; **p<0.01 or ns (p>0.05: not significant) analyzed in unpaired Student's t-test.
